# Determining the Reactivity and Titre of Serum using a Haemagglutination Assay

**DOI:** 10.3791/1752

**Published:** 2010-01-29

**Authors:** Maurizio Costabile

**Affiliations:** School of Pharmacy and Medical Sciences, University of South Australia

## Abstract

Haemagglutination is a specific form of agglutination and is used when antibodies bind to red blood cells, which act as a particulate antigen. Red blood cells are particularly useful targets as they are readily available and agglutination is observable using the naked eye. This technique is commonly used to determine the titre of an antibody (Ab), for blood grouping and viral quantification. In this video, the steps involved in preparing and performing a haemagglutination assay is demonstrated using antibodies specific to blood group A-antigens added to red blood cells (Revercells). The antiserum is serially diluted in a 96 well U-bottom microtitre tray, to which is added a suspension of Revercells. The samples are mixed and then incubated at 37°C for 60 minutes. After this time, the samples can then be easily scored for  ve, +ve and intermediate (-/+) haemagglutination reactions. This approach allows for the reactivity and titre of a serum sample to be assessed using a rapid and simple technique. The video will cover the theory behind the assay, how the results are read and interpreted, how the titre is determined, how the assay can be modified and any issues associated with the use of this technique.

**Figure:**
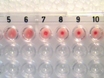


## Protocol

### Preparation of 5x Veronal Buffered Saline (VBS)

To prepare the Veronal Buffered Saline (VBS), three separate solutions need to be prepared.
Prepare solution 1 by dissolving 21.25gm of NaCl and 0.94gm of Sodium Barbitone in 350ml of distilled water. The final concentrations of the NaCl and Sodium Barbitone are 1.02M and 13mM respectively.
Prepare solution 2 by dissolving 1.44gm of Barbitone in 125ml of Hot distilled water. The final concentration of Barbitone is 62.5mM.
Prepare solution 3 by dissolving 20.33gm of MgCl_2_ and 4.41gm of CaCl_2_ in 100ml of distilled water. The final concentration of MgCl_2_ and CaCl_2_ is 2.18M and 440mM respectively.
Mix solutions 1 and 2 and cool to room temperature.
Once the combined solution has cooled, add 1.25 ml of solution 3 and adjust the pH to 7.3-7.5 using 1M HCl.
Adjust the final volume to 500ml with distilled water to prepare a 5x stock solution.
To prepare a 1x working solution, dilute the stock 1:5 with distilled water.

### Dilution of Revercells

Gently resuspend the stock solution of Revercells by inversion and then remove 1ml of cells and centrifuge for 5 min at 600g at 4°C.
After centrifugation, carefully remove the supernatant and resuspend the cells in 3ml of 1x Veronal Buffered Saline.
The cells can now be stored at 4°C until use.

### Procedure

Transfer 50ml of 1x VBS into rows A1-A12 of a 96 well U bottom plate.
To Well A1, add 50μl of anti-A serum and mix thoroughly using a pipette.
Using a fresh pipette tip, remove 50μl of liquid from well A1 and transfer it to well A2. Mix thoroughly and repeat this process until you reach well A11 and then discard the last 50μl from this well.
The serum sample has now been serially diluted 2-fold from 1:2 to 1:2048. No serum is added to Well A12 as this is the VBS negative control.
If more than one serum sample is to be assessed, repeat steps 4.1-4.4 for row B etc.
Once all the dilutions have been made, add 50μl of 1% Revercells to all wells. Note: The Revercells will settle over time and should be gently suspended prior to being dispensed into the wells.
Mix the samples by gently tapping on the side of the microtitre tray, cover and leave the haemagglutination reaction to proceed for 1h at 37°C. An incubator or appropriately heated room can be used.
Following incubation, carefully remove the tray from the incubator and examine the plate for haemagglutination. The VBS control well should be the first well examined. If the reaction is negative, then the results are valid. If the VBS sample shows a positive haemagglutination result, then the results cannot be used and the process should be repeated. A positive haemagglutination reaction will appear as a broad sheet on the base of the well, while a negative reaction will appear as a small concentrated pellet of cells in the centre of the well. An intermediate result will have a halo of positive cells with a central core of pelleted cells. This result occurs as the serum has some Ab that can react, but insufficient amounts of Ab to cause a full reaction.
The representative results section has two diagrammatic representations of the expected results. An example of an invalid and valid set of results is provided. The titre for the anti-serum will also be determined.

### Representative Results:

The video includes an example of representative results. Below (Fig1 and Fig 2) are two examples shown in a diagrammatic manner. The first example is of a reaction that cannot be interpreted as the VBS control is positive, while the second is a valid assay that allows for the titre to be determined.


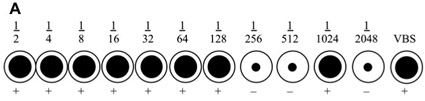
 **Figure 1.** An example of a haemagglutination where the positive result has failed. In the diagrammatic representation above, the heamagglutination reaction cannot be accepted. The VBS serum negative control displays a positive haemagglutination reaction as evidenced by the sheet formation at the base of the well. This indicates that an error has occurred and the results cannot be accepted. In this case, the process should be repeated.


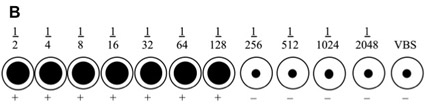
 **Figure 2.** An example of a haemagglutination where the positive result has worked correctly. In the diagrammatic representation, the heamagglutination reaction is valid. The VBS negative control displays a negative haemagglutination reaction as evidenced by the pellet that has formed at the base of the well. The last positive well is row 7, which indicates that the titre for this serum sample is 128 (i.e the reciprocal of 1/128). This would be an example of an antibody that is present in moderate amounts in the serum.

## Discussion

The haemagglutination assay is a very simple technique that allows for a large number of serum samples to be assessed within a short period of time. It also has the advantage of being read without the need for specialised equipment, with the eye being suitable. This technique can also be modified for the detection of human chorionic gonadotrophin hormone present in urine, as part of a haemagglutination-inhibition test. In relation to a standard haemagglutination test, there are a few areas where care needs to be taken in order for the assay to be performed correctly. Firstly, this technique is based on the settling of cells onto a concave surface. As such, it is essential that a U- or round bottom microtitre plate is used. The use of a flat bottom plate as is commonly used for ELISA is not suitable, as negative and positive reactions will appear identical. It should also be appreciated that when different serum samples are used, reactive antibodies are likely to be present at differing concentrations. At high antibody concentration, the antigen (Revercells) may be limiting and a prozone effect may be observed. However this will not affect the determination of the titre value, since this is determined as being the last well where a positive reaction was last seen. Lastly, this technique is not quantitative, the absolute level of reactive antibody in the serum is not known. However, in most cases this is not necessary, particularly when the determination of reactivity to a known antigen is all the information that is required.

